# Prosocial behavior in competitive fish: the case of the archerfish

**DOI:** 10.1038/s42003-023-05195-1

**Published:** 2023-08-08

**Authors:** Orit Nafcha, Dana Vilker, Simone Shamay-Tsoory, Shai Gabay

**Affiliations:** 1 School of Psychological Science, University of Haifa, Haifa, Israel; 2 The Institute of Information Processing and Decision Making (IIPDM), Haifa, Israel

**Keywords:** Human behaviour, Decision, Social behaviour

## Abstract

Humans are social creatures, demonstrate prosocial behaviors, and are sensitive to the actions and consequent payoff of others. This social sensitivity has also been found in many other species, though not in all. Research has suggested that prosocial tendencies are more pronounced in naturally cooperative species whose social structure requires a high level of interdependence and allomaternal care. The present study challenges this assumption by demonstrating, in a laboratory setting, that archerfish, competitive by nature, preferred targets rewarding both themselves and their tankmates, but only when the payoff was equal. With no tankmate on the other side of the partition, they exhibited no obvious preference. Finding evidence for prosocial behavior and negative responses to unequal distribution of reward to the advantage of the other fish suggests that in a competitive social environment, being prosocial may be the most adaptive strategy for personal survival, even if it benefits others as well.

## Abstract (web summary)

Archerfish, competitive by nature, are found to prefer targets that reward both themselves and their tankmates when the payoff is equal, providing an example of prosocial behavior.

## Introduction

Prosociality is defined as a low-cost behavior whose outcome produces benefits for others^1,2^. Over the past few years, numerous studies have demonstrated the gap between classic economic theory and prosocial human behavior. According to classic economic theory, decisions are motivated by self-interest without considering the interests of others^3,4^. Yet prosocial behavior in humans is very common and is manifested in a variety of ways^5–10^. Indeed, some have even suggested that it is a primary impulse for humans to cooperate^11^.

But is prosocial behavior unique to humans? Clearly it is not. Accumulating evidence points to prosocial behavior in animals, particularly in primates^12–15^ but also in parrots^16^, mice and rats^17–19^ and fish^20^. Inconsistent evidence (reviewed in^2,21,22^) suggests that phylogenetic closeness to humans is not conclusively associated with higher prosocial tendencies. Instead, allomaternal care has been proposed as an explanation to underlie prosociality;^20,23,24^ see^25^ for alternative theories. For example, based on data from a sample of 15 primate species, Burkart et al.^23^ extrapolated a positive correlation between allomaternal care and prosocial behavior. A related hypothesis proposes that interdependence is associated with prosociality, suggesting that prosociality is more prevalent in species with strong social interdependence^12,26,27^. According to this hypothesis, an agent will prefer making prosocial choices toward others when the benefits of these choices are positively associated with self-benefits^12,28^. Since species with highly developed allomaternal care also exhibit high interdependence among individuals^24,29^, both theories are highly correlated.

Recently, Satoh et al.^20^ demonstrated prosocial and antisocial behavior among the monogamous and biparental cichlid fish. They have found that male fish prefer prosocial choices when the recipient is their mate, or another female when their mate is absent, but they did not favor prosocial choices in other contexts. The researchers concluded that since the cichlid fish, which are monogamous with biparental care, examined in their study demonstrated prosociality, their findings strengthen the allomaternal care hypothesis^23^ and the interdependence hypothesis. Notably that prosocial behavior could emerge also through other mechanisms such as reciprocity^25,30^. Apart from selection theories, the observed prosocial behavior which involves no costs for the agent may also be developed and spread through evolution by genetic drift^31^.

Here we asked whether prosocial behavior is unique to naturally social and collaborative species with strong positive interdependence or whether it is also present in more competitive species with no grouping preference or monogamous mates. Is interdependence of any type a necessary condition for prosociality to emerge?

To address this question, we chose to explore whether sensitivity to another’s reward, as manifested both in prosociality and in aversion to unequal distribution of reward to the advantage of another fish, is evident in fish that live in a competitive social environment. In addition, we wanted to explore whether fish whose neural systems are relatively less complex than those of mammals^32^ and who lack neocortex-like cells would present deliberate prosociality in a laboratory setting. We have focused on the competitive archerfish as will be described in what follows.

Comparing fish cognition and behavior to that of primates has many challenges. Most studies have used different experimental manipulations and different dependent variables when studying non-primates. We specifically selected archerfish (*Toxotes chatareus*) to serve as our model because of their remarkable ability to shoot down insects found on foliage above water level^33–35^. This ability can be exploited by having archerfish complete computerized tasks in a laboratory setting, similar to humans and primates. Instead of pressing a button to choose a target (as is the case with humans and primates), archerfish spit at the target. This allows us to use the same tasks and manipulations to explore and compare fish and human cognition (for further details, see ref. ^36^). This model has already been employed to study such factors as attentional orienting^37,38^, face discrimination^39^, visual search^40^, social cues^41^, learning and generalization^42^, and decision-making^43^. Archerfish have the ability to learn to distinguish between artificial targets shown on a computer monitor in an experimental setting^39^. The fish’s ability to participate in such controlled and complex experimental procedures provides a unique opportunity to uncover whether cortical brain structures are a prerequisite forprosociality tendencies to emerge.

One feature that makes archerfish particularly ideal for studying social interactions is that when first learning to shoot, they hunt in small schools, thus requiring young archerfish to interact socially. Yet, foraging socially in the wild also encourages intraspecific cleptoparasitic behavior, compelling archerfish to compete directly (and often aggressively) for the prey shot down by other archerfish^35,44,45^. This competitive social environment requires sophisticated hunting skills^36,45,46^, together with better predictive ability regarding both the action outcomes^47^ and the behavior of others. Hence, perception and imitation of others are essential properties of archerfish cognition. Indeed, previous studies have demonstrated that archerfish can learn complex sensorimotor skills simply by observing the actions of other group members, without the need to practice themselves^42^. Some studies^48^ even found that archerfish are sensitive to the presence of an audience while foraging (e.g., the audience has an impact on how long it takes archerfish to shoot, but also see^49^, which did not find any effect of the social context on archerfish learning rates). Establishing whether the competitive archerfish exhibit prosocial behavior could shed light whether even a competitive species, with no grouping preference^34^, would demonstrate this kind of behavior.

Research has used three main procedures to test prosociality in nonhuman primates: the prosocial choice task, the food sharing task, and the targeted-helping task^24^ (p.199); see also refs. ^15,22^. In the present study we use the prosocial choice-based task (PCT)^14,15,22,50–55^. In this task, one subject is given a choice between two actions that require equal effort but differ in the outcome for a passive partner, while not differing in the outcome for the agent. This task is typically compared with a control task in which there is only one agent and no other potential recipient. In addition to examining social tendencies, this choice-based procedure makes it possible to compare animals’ preferences for equal versus unequal reward distribution without requiring the agents to sacrifice their own outcome.

In the current study, we used a choice-based task in which the outcome for the acting fish always remained constant (currently, one food pellet), allowing us to control for nonsocial alternative explanations (for instance loss aversion)^55^. In the first experiment, the acting fish was required to choose between two options—an unequal reward distribution that is advantageous to the acting fish (i.e., non-prosocial), leaving its tankmate with no food (1/0), or an equal reward distribution (1/1) between the agent and its tankmate (i.e., prosocial). After obtaining results of the first phase in which the fish chose significantly more the social outcome targets, we wanted to make sure that this preference was not a result of target properties. This led us to conduct a second phase of the experiment in which we reversed the color mapping. After establishing that the results were not a product of target properties, we wanted to examine whether the fact that more food in general was supplied after a social choice did not influence our results. In order to explore this possibility, we conducted the second experiment which was the same procedure as the first one but without a fish on the other side of the partition. A third experiment was aimed at exploring the limits of the prosocial tendencies of the fish by creating an unequal distribution of food for the social target to the disadvantage of the acting fish. Concretely, the acting fish was required to choose either an unequal reward distribution that was to its advantage (1/0) or an unequal reward distribution that was to the benefit of its tankmate (1/2). As can be seen in the Results section, our findings imply that even archerfish, which are competitive by nature, exhibit prosocial behavior so long as no advantage is given to the other fish.

## Results

### Experiment 1: Prosocial experiment

**Fig. 1:**
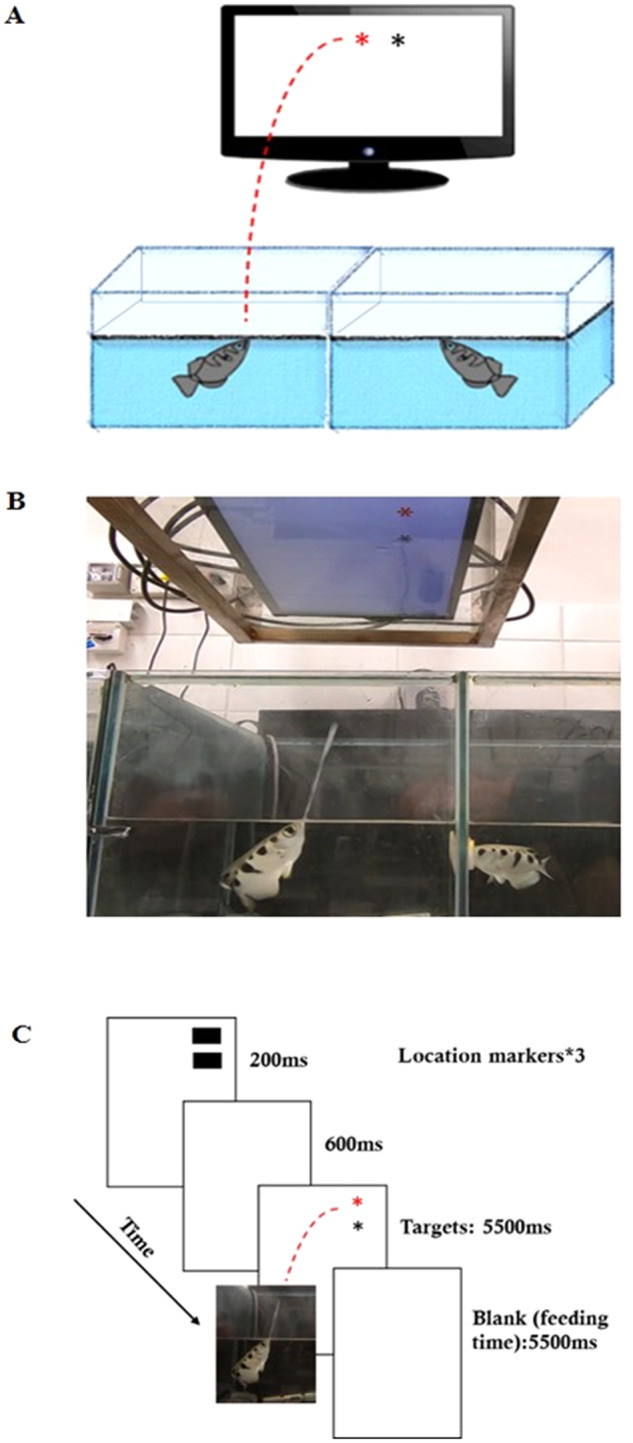
Experimental setup and trial sequence. Experimental set-up and trial sequence. **A** illustrates the setup. There was a dual tank in which two fish shared the same water environment and could see each other but were separated by a partition that prevented food or fish from moving from one side to the other. The acting fish was required to choose between two targets, presented on a computer screen positioned above it. The two targets (prosocial, non-prosocial) differed in their outcome for the receiving fish. **B** is a photo of the actual set-up and a demonstration of the fish spitting in the laboratory setting. **C** shows the sequence of events in a typical trial. At the beginning of the trial, two black location markers flickered for 200 ms. After a 600 ms blank interval, the markers appeared again. This was repeated three times. The targets then appeared at one of three locations for 5500 ms (see Method section). After the fish spat or the target interval ended, a blank screen was shown for 5500 ms before the next trial began. At this time, the fish received the outcome according to its choice of target.

In the current experiment, fish were required to choose between stimuli whose associated outcomes were either prosocial (equal distribution) or non-prosocial (unequal distribution). In the first phase, the fish was presented with two targets of different colors. Responding to one target (e.g., black asterisk; see the Supplementary Table 5 for a description of which colors were used for each fish separately) resulted in one food pellet given only to the acting fish (1/0). Responding to the other target (e.g., red asterisk) resulted in one food pellet given to the acting fish, but also one food pellet given to the fish on the other side of the partitioned tank (1/1). In both cases, the outcome for the acting fish was the same. In the second phase, in order to rule out any possible effect of target properties (e.g., target color), we changed the mapping between target color and reward type, such that the target color originally associated with the equal distribution (prosocial) outcome was now associated with the self-advantage (non-prosocial) outcome and vice versa. Note that in all the experiments, the fish shared a dual tank, hence the same water environment, yet they could not move from one side of the partition to the other and the food was delivered to one or both sides in response to the agent fish’s choice (see Fig. 1 for illustration, and Supplementary Movie 1 and the Method section for further descriptions).

We conducted the experiment on four fish as agents who had to choose between the targets and four fish as passive observers whose outcome was dependent on the choice made by the agent. Three fish served as agents only and three other fish served as receivers only. An additional two fish acted as an agent and a receiver in one experiment and then reversed roles in another experiment at a different time. Over all, a total of six fish participated in the experiment. See Supplementary Table 1 in the supplemental material. The sample size we used is common in archerfish studies (see refs. ^37–39,41,48,56^). Although we used a relatively small sample, each fish performed a large number of trials (for a similar approach from studies on human cognitive abilities see refs. ^57–59^). Note that Fish 2 and 3 were familiar with each other since they had done a previous (not pro-social) study together, while Fish 1 and 4 each acted with a new partner. The previous acquaintance of 2 and 3 was pro-socially irrelevant for fish 2 and it did not make a difference between its results and the others. Fish 3 however as the receiver of fish 2, did have pro-social relevant acquaintance with fish 2, which may be related to its very pro-social learning and performance as you may see in the results. See the discussion of the first experiment.

Each session contained 40 trials in which the acting fish were required to choose one of the two targets. The outcomes associated with each target were different for the passive fish yet remained the same for the acting fish. Throughout the experiment (both phases), each fish retained its active or passive role so there were no reciprocal relations. The purpose of this was to rule out the possibility for other motives to evolve, such as a reciprocal strategy^16,60^. The target pair could appear at one of three different possible locations and the locations of the targets within the pairs were counterbalanced (see the Method section for further details on the experiment).

#### Results of experiment 1

**Fig. 2:**
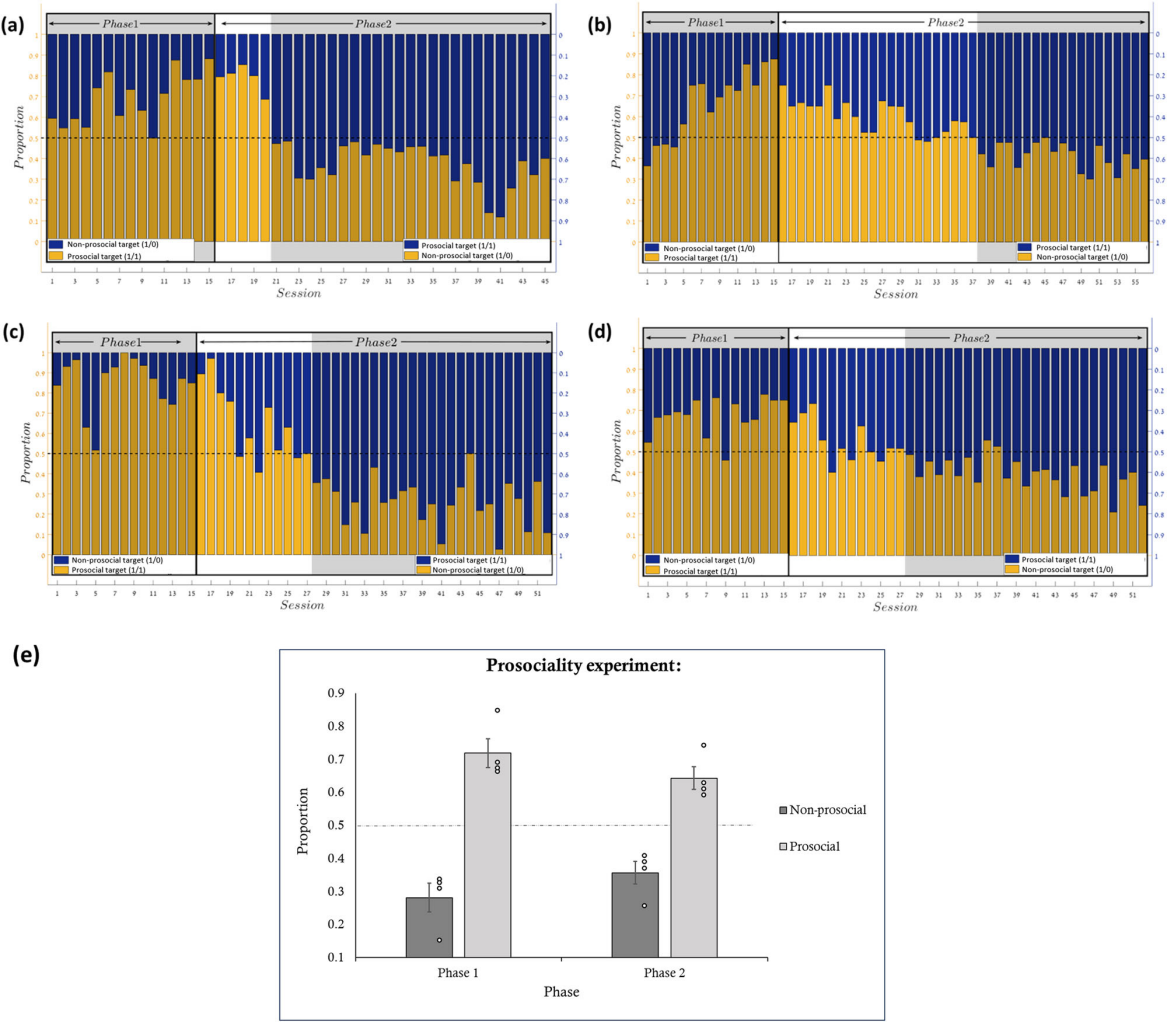
Results for four fish in the prosocial experiment (phase 1 and phase 2). Results for four fish in the advantageous experiment (phase 1 and phase 2). The pattern of results (Phase 1 and 2) examining the fish’s preference for responding to targets that also provided a food pellet to a neighboring fish (equal distribution, prosocial) versus targets that provided the same reward for the acting fish but did not provide food for its counterpart (non-prosocial). **a**–**d** show the fish raw data, with each graph representing a different fish. Both Y axes represent the proportion of target selection, with the left-hand scale numbered for the yellow color and the right-hand scale for the blue color. The X-axis represents the number of sessions. The middle line represents 50% chance. The left side of each graph shows the first phase, in which the yellow bars represent the proportion of preference for the equal distribution (prosocial) target, resulting in a social outcome (1/1), and the blue bars represent the proportion of preference for the self-advantageous target, resulting in a non-prosocial outcome (1/0). In the second phase, the color mapping was reversed. The bars add up to 1. The gray area of the second phase represents the sessions that were included in the statistical analysis after the switch. **e** represents a summary across all fish for each target type selection in both phases (i.e., social, and non-prosocial targets). Error bars represent one standard error. The data points represent the average preference of each fish in each condition. Over all, the fish showed a significant preference for the equal distribution (prosocial) targets, regardless of color.

For the statistical analysis, in each phase we calculated the proportion of social target color choices in each session and then used one-sample *t*-test to compare the choices made by the fish to chance. Each session contained 40 trials. The fish did not necessarily spit in all 40 trials; hence the proportion was calculated from the valid trials (i.e., from the sum of both colors). If a fish did not spit in more than half a session, we omitted that session. See Supplementary Note 7 in the supplemental material. We also calculated Bayes factors (BF) using the free software JASP (https://jasp-stats.org). Note that in the second phase the criterion for learning was that the fish chose the new equal distribution (prosocial) color more than the old equal distribution color for three consecutive sessions (the second phase analysis included these three sessions). Please note that the difference from 0.5 is still significant even when analyzing all the trials, including those before the switch point, see the Supplementary Note 4 for the analyses. The first phase contained 15 sessions. The statistical analysis of the second phase (defined by the above criterion) contained 25 sessions, except in the case of Fish 2, for which phase two was terminated sooner (*n* = 19) for technical reasons (see the Method section for further details). The results of the first phase indicated that the acting fish preferred targets that provided food pellets also to its tank-mate [*t*_(14)_ = 5.96, *p* < 0.001, *d* = 1.54, *BF10* = 732.3; *t*_(14)_ = 3.83, *p* = 0.002, *d* = 0.99, *BF10* = 23.02; *t*_(14)_ = 10.05, *p* < 0.001, *d* = 2.6, *BF10* = 160028.32; *t*_*(14*)_ = 7.4, *p* < 0.001, *d* = 1.91, *BF10* = 5906.67; for fish 1–4 respectively]. This pattern of results was demonstrated also in the second phase [*t*_(24)_ = 6.4, *p* < 0.001, *d* = 1.28, *BF10* = 14176.26; *t*_(18)_ = 6.42, *p* < 0.001, *d* = 1.47, *BF10* = 4308.53; *t*_*(*24)_ = 10.31, p < 0.001, *d* = 2.06, *BF10* = 38580000; *t*_(24)=_6.51, *p* < 0.001, *d* = 1.3, *BF10* = 18417.94; for fish 1–4 respectively]. Over all, the results from all the fish revealed that in both phases the archerfish significantly preferred prosocial targets that resulted in equal distribution of the reward, regardless of the target properties (phase 1- data from all fish: *t*_(59)_ = 11.29, *p* < 0.001, *d* = 1.46, *BF10* = 2.790e + 13, phase 2–data from all fish-*t*_(93)_ = 12.79, *p* < 0.001, *d* = 1.32, *BF10* = 1.396e + 19). See also Supplementary Note 10 for further group analyses and Supplementary Fig. 1 (in Supplementary Note 8). That is, the fish preferred targets that led to outcomes which also benefited their tank-mate. See Fig. 2a–e, Supplementary Note 1, and Supplementary Table 2 in the supplemental material for the descriptive statistics.

A closer inspection of the pattern of results for the one fish that acted first as a receiver and then as an agent (Fig. 2c) reveals that this fish demonstrated faster learning of the regularities (the outcome for each target) in both phases of the experiment. These findings correspond to previous evidence^42^ demonstrating that fish can learn sensorimotor skills just by observation. The current pattern might suggest that abstract social rules can also be learned just by observation; see also^61^, which demonstrated that previously soaked rats were quicker to help free other soaked rats. Whether fish can actually learn social regularities just by observation is beyond the scope of the current study and indeed this finding may also be incidental. This question should be directly tested in future studies.

### Experiment 2: Control experiment

The results of the first experiment (both phases) revealed that the fish exhibited prosocial tendencies when the alternative was an unequal situation to its advantage. Nevertheless, one may argue that the fish’s preference for the prosocial targets is a consequence of delivering two pieces of food (one on each side of the partition) rather than one (only on the side of the acting fish), regardless of the social outcome. This preference may be explained in two ways: either as a means of increasing the overall amount of food delivered (regardless of its accessibility) or as a reinforcement of agency, where two events occurring as a result of the agent’s action provide greater control feedback^62^. The main point of this alternative explanation is that the food dropped into the other side of the tank influences the acting fish, regardless of the presence of the passive fish. In order to rule out this possibility, as was done previously when using a similar social task^22^, we conducted a second experiment in which the reward mapping was identical, with the only difference being that there was no fish on the other side of the partition. In this control experiment, choosing one color resulted in one food pellet given to the acting fish (a single event), while choosing the other color target resulted in one food pellet given to the acting fish but also one food pellet dropped into the other side of the partition, where there was no fish (dual events). Note that as in the first (social) experiment, every session consisted of forty trials in which the acting fish was required to choose. To rule out the possibility that the results were related to the stimuli properties (target color), the reward mapping was reversed in the second phase. So that this control experiment would resemble the first experiment as closely as possible, the food dropped into the empty side of the tank was pulled out of the water during the experiment while the fish was consuming its own reward (see Method, supplemental material and Supplementary Movie 2).

This experiment was conducted on four fish. One, however, did not meet the criterion for accurate hits (see Method section for further details) and therefore was not included in the main analysis. The results for this fish, which resembled those of the other control fish, are discussed in Supplementary Note 2 in the supplemental material. The same fish participated both in the control and in the first (prosocial) experiment, which were separated by another experiment. This was true for all three fish included in the analyses. The fish excluded for failing to hit the targets accurately participated only in the control experiment.

Note that although this experiment was not conducted immediately after the first experiment, the colors were changed in order to rule out any order or carry-over effect. For the full-color table see Supplementary Table 6 in the supplemental material.

#### Results of experiment 2

In the statistical analyses, for each phase we calculated the proportion of dual-event target choices in each session and compared the fish’s choices to chance by using a one-sample *t*-test. We also calculated Bayes factors (JASP; https://jasp-stats.org).

**Fig. 3:**
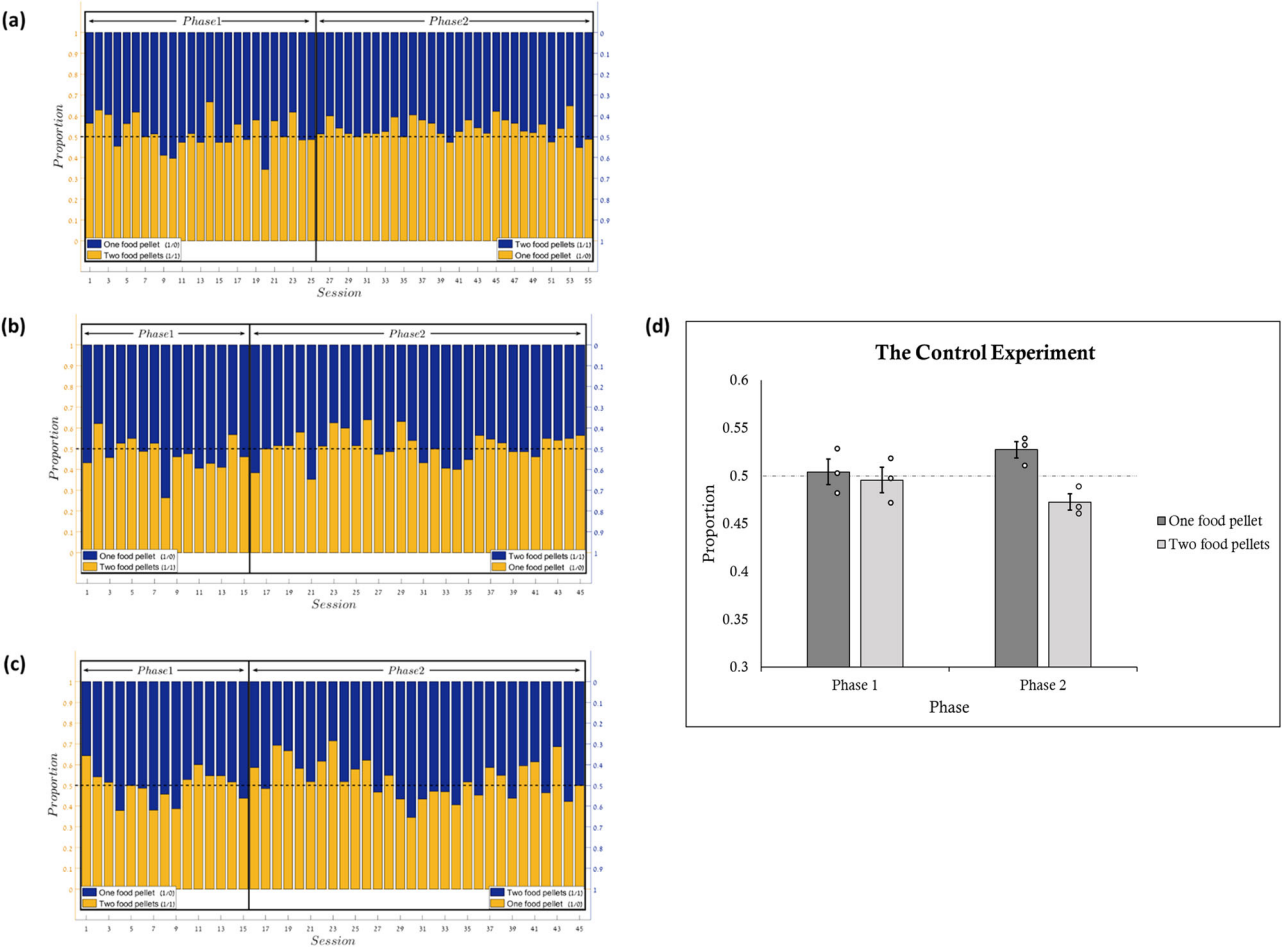
Results of the control experiment (phase 1 and phase 2). Results of the control experiment (phase 1 and phase 2). This figure shows the pattern of results (Phase 1 and 2) examining the fish’s preference to respond to targets that also provide a food pellet for an empty side (two pellets) versus targets that provide the same reward for the acting fish but do not provide food for the other side (one pellet). **a**–**c** show the fish raw data, with each graph representing a different fish. Both Y axes represent the proportion of target selection. The X-axis represents the number of the session. The middle line represents 50% chance. The left side of each graph represents the first phase, where the yellow bars represent the proportion of preference for the dual events resulting in two food pellets (1/1) and the blue bars represent the proportion of preference for the single event, resulting in one food pellet (1/0). In the second phase the color mapping was reversed. The bars add up to 1. **d** represents a summary across all fish for each target type selection (i.e., dual events, single event). Error bars represent one standard error. The data points represent the average preference of each fish in each condition. Over all, no significant preference emerged for the dual events when there was no fish on the other side of the partition.

The results of the first phase revealed that when there was no fish on the other side of the partition there was no preference in the acting fish’s selection [*t*_*(24)*_ = 1.15, *p* = 0.26, *d* = 0.23, *BF10* = 0.38; *t*_*(14)*_ = 1.3, *p* = 0.21, *d* = 0.34, *BF10* = 0.53; *t*_*(14)*_ = 0.13, *p* = 0.9, *d* = 0.034, *BF10* = 0.26; for fish a-c respectively]. This pattern remained for two fish (Fig. 3b, c) that exhibited no statistically significant difference in the selection of the two targets in either phase. This pattern was also observed for the fish we excluded from the main analysis (see Supplementary Note 2). The remaining fish (Fish 1, Fig. 3a) exhibited in the second phase a statistically significant preference for the single-event target, the one that provided food only on the fish side [*t*_*(29)*_ = 4.56, *p* < 0.001, *d* = 0.83, *BF10* = 303.65 (preference for a single event); *t*_*(29)*_ = 0.82, *p* = 0.41, *d* = 0.15, *BF10* = 0.26; *t*_*(29)*_ = 1.87, *p* = 0.07, *d* = 0.34, *BF10* = 0.9; for fish a-c respectively]. Overall, when there was no fish on the other side of the partition there was no difference from chance in both phases (phase 1 all fish–*t*_*(54)*_ = 0.02, *p* = 0.98, *d* = 0, *BF10* = 0.14, phase 2 all fish - *t*_*(89)*_ = 3.5, *p* < 0.001, *d* = 0.37, *BF10* = 33.3 [M p(1/0 > M p(1/1)]). When analyzing the results from three fish that participated in both experiments (the social and the control) we found that the presence or absence of a fish on the other side made a significant difference in the fish preference for choosing the two-pellet target. See Fig. 3a–d and the Supplementary Note 3 for a paired t-test for each fish examining the difference in the proportion of choosing the social over the non-prosocial target between the social and the control experiments (only for the three fish that participated in both experiments). See also Supplementary Table 3 for the descriptive statistics.

### Experiment 3: Unequal reward distribution experiment

In the first experiment, we explored whether fish prefer situations that give them an advantage over other fish or rather avoid such situations by choosing an equal distribution of reward, thus making prosocial choices by choosing targets that equally reward themselves and a partner. With Experiment 3, we strove to test the limits of prosociality and to explore whether the acting fish would still favor targets that also benefitted its tankmate when the tankmate received more food than itself. Essentially, we examined how fish react to an unequal situation which is to the advantage of the other fish.

In the current study, the reward for the acting fish was kept constant while the outcome for its tank-mate was manipulated. The acting fish was required to choose between a target that rewarded only itself with one food pellet (1/0) or a second target that still rewarded itself with one food pellet but also delivered two food pellets to the passive fish (1/2). Given the competitive nature of the archerfish^44,45,48^, we predicted that the result of unequal distribution of rewards would be that the acting fish would no longer choose the target that benefits both fish so as to prevent giving an advantage to the other fish, as it would increase its own perceived vulnerability^44,63^.

**Fig. 4:**
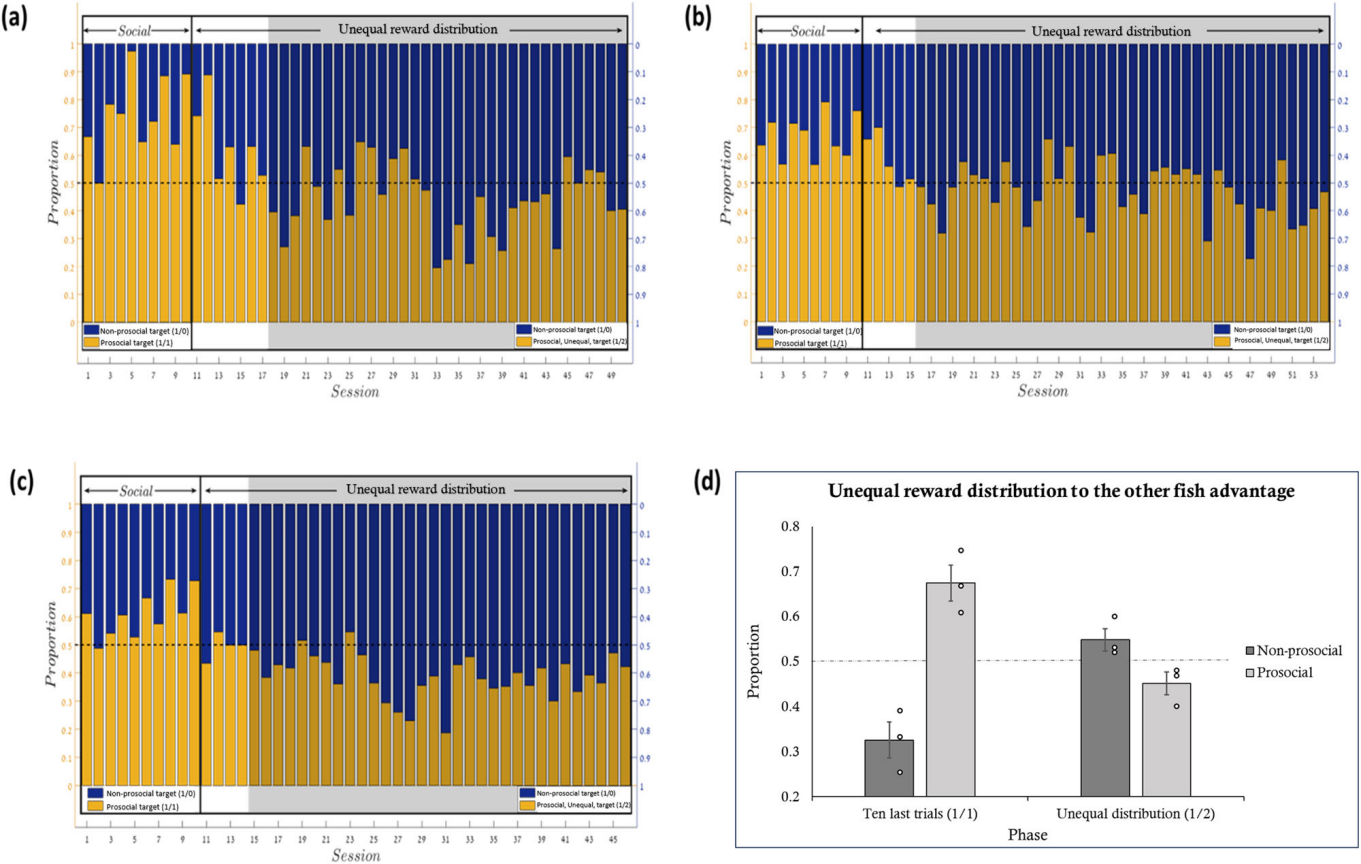
Results of the third experiment, unequal reward distribution. Results of the third experiment. The pattern of results of the fish’s preference for targets providing food only to itself (non-prosocial targets) or for targets providing a food pellet to the advantage of a neighboring fish (1/2) (unequal prosocial). **a**–**c** show the fish raw data, with each graph representing a different fish. Both Y axes represent the proportion of target selection, with the left-hand scale numbered for the yellow color and the right-hand scale for the blue color. The X-axis represents the number of the session. The middle line represents 50% chance. The left side of each graph shows the last ten sessions of the first prosocial experiment in which the prosocial target benefiting both is associated with equal distribution of rewards (1/1). The yellow bars represent the proportion of preference for the equal distribution (pro-social) target, and the blue bars represent the proportion of preference for the non-prosocial target. In the second phase (the current experiment), the yellow bars represent a social outcome to the other fish advantage (1/2) and, as before, the blue bars represent the proportion of preference for the non-prosocial target (1/0). The bars add up to 1. **d** represents a summary across all fish for each target type selection in both phases. Error bars represent one standard error. The data points represent the average preference of each fish in each condition. Over all, the fish stopped choosing the target that resulted in a social outcome when the passive fish received more food than the acting fish.

This experiment was conducted on three fish as agents and another three fish as passive receivers. The experimental procedure resembled that of Experiment 1 (1/0, 1/1), with the sole exception that spitting on the social target resulted in two food pellets given to the passive fish (1/0, 1/2). Note that the first two acting fish (Fig. 4a, b) participated in the first social experiment (Fish 3 and 4 in experiment 1) in its entirety and immediately afterward participated in this experiment so that we could observe whether and how their preference changed. A third acting fish (Fig. 4c) was run specifically for this experiment and hence performed only the first phase of the prosocial experiment, without the switching phase (phase 2 of experiment 1) and then reward mapping shifted to the unequal distribution which is to the advantage of the other fish. See Supplementary Tables 1, 7.

#### Results of experiment 3

In the statistical analyses, we calculated the proportion of the self-disadvantage target choices (1/2) in each session and compared the fish’s choices to chance by using a one-sample *t*-test. We conducted this analysis twice: a) for all the sessions after the outcome changed from equal distribution (1/1) to self-disadvantage (1/2); b) for the sessions from when a switch point was detected (similar to the analysis of the second phase of the first experiment, in which a ‘switch’ was determined to have occurred after three consecutive sessions of more than 0.51% preference change). We also calculated Bayes factors (JASP; https://jasp-stats.org). Analysis of the last ten trials of the prosocial experiment demonstrated that when the outcomes for both fish were equal (1-1) the fish favored the equal distribution targets [*t*_*(9)*_ = 5.47, *p* < 0.001, *d* = 1.7, *BF10* = 89.05; *t*_*(9)*_ = 6.72, *p* < 0.001, *d* = 2.12, *BF10* = 323.15; *t*_*(9)*_ = 4.2, *p* = 0.002, *d* = 1.33, *BF10* = 20.7; for fish a-c respectively and *t*_*(29)*_ = 8.19, *p* < 0.001, *d* = 1.5, *BF10* = 2.542e + 6 for all fish together]. When the passive tank-mate received more food than the active fish (1/2) the agent fish stopped choosing the target that benefitted both fish [*t*_*(39)*_ = 1.31, *p* = 0.2, *d* = 0.2, *BF10* = 0.38; *t*_*(43)*_ = 1.24, *p* = 0.22, *d* = 0.18, *BF10* = 0.33; *t*_(35)_ = 7.26, *p* < 0.001, *d* = 1.21, *BF10* = 759033.79 (preference for self-advantage target); for fish a-c respectively and *t*_*(119)* _= −4.3, *p* < 0.001, *d* = −0.39, *BF10* = 464.07 for all fish together]. Furthermore, when analyzing only the sessions from a switch point, similarly to the first experiment, the fish demonstrated a significant bias toward the self-advantage distribution target [*t*_*(32)*_ = 2.83, *p* = 0.008, *d* = 0.49, *BF10* = 5.32; *t*_*(38)*_ = 2.03, *p* = 0.049, *d* = 0.33, *BF10* = 1.1; *t*_*(31)*_ = 8.03, *p* < 0.001, *d* = 1.42, *BF10* = 2752000 for fish a-c respectively; and *t*_*(103)*_ = −6.25, *p* < 0.001, *d* = −0.61, *BF10* = 1.167e + 6 for all fish together]. See Fig. 4a–d and Supplementary Table 4 in the supplemental material for the descriptive statistics.

## Discussion

In the present study we examined in the archerfish, a competitive species, two questions: a) whether the fish manifest prosocial tendencies in favoring targets which benefit other members as well as the agent, and b) whether the fish are averse to unequal distribution of reward in favor of another fish. In the first two experiments, the fish chose the prosocial targets that rewarded both fish, but only when there was a fish on the other side of the tank. In the third experiment the fish refrained from choosing targets that would result in unequal distribution that was to the passive fish’s advantage.

In nature, archerfish are faced with a competitive environment (inter- and intra-specific)^44,45^. The laboratory setting of the current study offers a novel demonstration of sensitivity to the social distribution of outcomes, which in some cases is manifested as prosociality. One possible explanation for prosociality in archerfish, known for their competitiveness, is that it serves as a means of avoiding punishment and condemnation (see also refs. ^64–66^). It may be the best strategy for the agent fish to choose the target that results in a prosocial outcome in order to avoid violent situations and not to choose this option if perceived as vulnerable. The present study reinforced this hypothesis through the fish’s more frequent tendency to choose the target that provides food for a neighboring fish, regardless of target properties. This pattern of behavior was not found in the absence of a partner fish or when the other fish received more food than the acting fish. Hence, in competitive environments or when food is limited, prosocial behavior could be the best strategy for long-term individual survival. This type of prosocial behavior could, of course, also be developed in cooperative environments but through different mechanisms.

Prosociality could develop with non-kin via reciprocal relationships (direct reciprocity through experience or indirect reciprocity through observation)^25,30^. Please note that even though actual reciprocity was not part of the study procedure, we cannot rule out that the fish choices were driven by their perception of potential future reciprocity. Furthermore, it has been suggested that if a behavior involves no costs, its underlying decision rule may spread in a population by random drift, which may provide a basis for generalized cooperation mechanisms to evolve^31^. Notably, according to generalized reciprocity, one helps another having been helped. While in the current study, there was no option for the passive fish to respond to the agent, generalized reciprocity may still explain these results if the rule applied also to potential situations in the future where the fish would choose prosocially as a general decision rule-based, not only on the past but also on the future: "help if you might be helped in the future". This is also in line with the motive to help from reputational benefits or future gain. We should also point out that fish may choose prosocially to reduce aversive arousal that may result from sensing others in need^67^. Archerfish live in a group and interact with multiple partners. Nevertheless, given that their social dynamic is determined by competition, changing partners may not be costly. A previous study found social choices in the cichlid fish and attributed this finding to the monogamous nature of this species^20^, reinforcing the hypotheses that either allomaternal care or strong positive interdependence are at the root of prosociality. Our study calls these hypotheses into question by demonstrating that prosocial behavior and a negative response to an unequal situation occur even in a competitive species with a low/negative level of interdependence or allomaternal care.

Previous studies have demonstrated that social behavior in mammals may be modulated by social aspects of the co-actor. Horner et al.^15^ found that chimpanzees are pro-social toward all partners, while the pro-social tendencies of Capuchin monkeys are dependent on social closeness^68^. Rats as well differentiate their social behavior relative to the social affiliation of their partner (e.g., kin, life-long group members^69,70^; see also ref. ^2^). In the current study, all the fish belonged to the same species (Toxotes chatareus) and shared the same water environment. In order to explore whether the observed sociality is selective and what the role of different social factors is, this paradigm should be examined when the two fish belong to two distinct species. In addition, it should be noted that the current study employed a relatively small sample size, hence future studies should examine whether the prosocial tendencies shown in the current study are replicated in larger samples.

An alternative explanation for the archerfish’s preferences for prosocial choices found in the first experiment may be that this preference is a way to distract the tankmate from concentrating on the agent’s reward. In nature, increased attention focused on the hunting fish can result in stealing its catch. Although the partition between the fish prevented the passive receiver from stealing the agent’s food, the acting fish may still have preferred a situation in which it received its reward while its tankmate was itself engaged in receiving food. Nevertheless, the findings of Experiment 3 provide evidence that weakens this possibility. According to this hypothesis, the tankmate should be more distracted as the amount of food is increased. Yet the acting fish did not favor the social target, thus reducing the likelihood of this explanation.

In the current study, we used a choice-based task in which the effort required and the subsequent outcome for the acting fish both remained constant throughout the experiment. Keeping the outcome identical for the acting fish regardless of its choice excluded the influence of any previous experience the fish may have had with different reward amounts. This invariability in the agent’s outcome allows us to rule out nonsocial motives previously suggested to explain the results of studies examining prosociality and fairness (for a review see ref. ^55^). Over all, the results of this study reinforce the notion of a social motive for the acting fish’s preference and aversion to a reward discrepancy between itself and its tankmate when it is to the other fish’s advantage.

Furthermore, scholars have claimed that our need to cope with a complex social environment is the cause of the evolutionary development of our large brains^71^ (but see ref. ^72^). Most of the current neuroscientific literature tends to attribute high cognitive abilities to brain areas that are highly developed in humans (i.e., cortical regions) rather than to neural substrates that are shared across multiple species (i.e., subcortical regions)^73^. The human cortex is also considered to be involved in many social processes. Our results provide additional evidence that having a cortex is not a necessary condition for prosociality, see also ref. ^74^ for a review on homologous brain structures across species. In this regard, note that we are not claiming that the brain regions responsible for prosociality in humans are the same in other species (e.g., fish). Indeed, it has been suggested that cortical regions may take control over subcortical mechanisms in order to develop new cognitive abilities. This lower neural circuitry may be recycled and adjusted to enable different cognitive functions to evolve^75–77^. Nevertheless, it plays an essential role in the evolutionary development and execution of many social abilities.

To conclude, to the best of our knowledge this is the first laboratory study to provide evidence for deliberate prosocial behavior and negative responses to an unequal situation in a competitive fish. Moreover, while the goal of gaining a positive interdependence may be the dominant mechanism leading to prosocial behavior in collaborative environments, in competitive environments an individual may choose to act in a prosocial manner in order to survive. Finding prosociality tendencies even in a competitive fish highlights the importance of this faculty and may reinforce the theory that sociality is intuitive^11^.

## Materials and methods

All our data were collected in accordance with the University of Haifa’s ethical standards and the State of Israel’s laws on animal care and experimentation. At no phase were the fish deprived of food or hurt in any way. In the first and third experiments, we tested whether the archerfish reacts to an unequal situation that is to its advantage or disadvantage and whether the fish shows any behavioral evidence indicating that it possesses prosocial tendencies. In each experiment, two fish were placed in a double tank and shared the same water environment, each on a different side of a partition. Each fish swam freely on its side of the partition, which did not allow the fish to pass from one side to the other, such that the fish could receive food only on its side of the partition. The control experiment (Exp.2) included one agent fish and no accompanying fish.

During the task, a 21-inch Samsung LCD monitor (model S24C650PL) was placed face down over the water, resting on a glass shelf 41 cm above water level; see Fig. 1. The fish were trained to shoot at the target stimuli and were recorded using a GigE Camera color (120 fps 640 × 480 1/4) and a GoPro HERO7. Shots were determined to be successful if the water jet hit any target presented on the screen. After each successful shot, food pellets (Tetra Discus Granules, Tetra Spectrum Brands Pet LLC, Virginia USA) were delivered from above according to the target mapping, and the experimenter cleaned the water off the glass shelf.

Each session contained 40 trials. Sessions in which a fish did not spit during more than 20 trials (half of the trials) were removed from the analysis (see Supplementary Note 7).

### Color preference

Before experimentation, each fish performed a color preference test to verify that it did not have any primary color preference or aversion (see Supplementary Note 5 for further details). In this color preference task, the fish received food for any shot, regardless of the target color. Following the color preference procedure, the colors we used for the experiment did not arouse significant aversion or bias. Moreover, note that color preferences cannot account for our results as we also employed a reversal phase. In the first experiment, we used red and black targets for Fish 1 and Fish 3 and green and black targets for Fish 2 and Fish 4 (see Supplementary Note 5 for the color allocation in each phase for each fish). In the control experiment (Exp. 2), we used blue and green targets for Fish a and b and black and green targets for Fish c. Note that fish color perception is similar to that of humans^36,78^. Nevertheless, in order to rule out the possibility that the fish could not distinguish between blue and green, we conducted a control experiment in which a fish in a single tank successfully distinguished between those colors when they were associated with different reward mappings. That is, spitting on the blue target resulted in food, while no food was delivered when the fish spat on the green target (see Supplementary Note 6).

### Stimuli and procedure

At the beginning and the end of each session, both fish received several food pellets as part of a fish-experimenter bonding ritual. At the beginning of each trial, the acting fish tended to swim near the water surface in expectation of target presentation. The passive fish almost always stayed by the partition. When the target was presented, the acting fish initiated its response by elevating its mouth above water level and shooting a stream of water at the chosen target (see Supplementary Movies 1, 2).

Stimuli were presented using E-prime 2 software. Each trial began with the flickering of two black location markers (3.49° height and 4.88° width), whose centers were positioned 9.89° from each other. The markers appeared for 200 ms at a time, with a 600 ms interval between appearances. After the location markers disappeared, two targets (asterisks with a radius of 1.39°) appeared for 5500 ms. The targets were red, green, black, or blue, depending on the condition and the fish, as described in note 5 in the supplemental material. The targets appeared at the same locations where the flickering black location markers had appeared. For Fish 4 (Fig. 2d), the markers and the targets were presented with their centers positioned 13.21° from each other. This is because the intensity of Fish 4’s spit was very strong, such that when the targets were too close, it was difficult to decide which target the fish had chosen. After the target disappeared, a blank interval screen was presented between trials for 5500 ms. During this time, the fish received food according to the choice made by the agent fish, and the experimenter cleaned the screen. Each fish performed one or two sessions every day. Two targets appeared as a pair 9.41° vertically from the center of the screen toward the partition, placed randomly in one of three possible locations that were distributed horizontally (see Fig. 1). The position of each target’s color in the pair was also randomized.

### Procedure

#### Experiment 1

Before each phase began, Fish 1 and Fish 2 underwent a learning procedure in which only one target was presented. We presented the fish with 32 trials in each session−16 showing only one color and 16 only the other color. For hitting each color, the acting fish was rewarded with the associated outcome. The fish were thereby introduced to all reward possibilities. This procedure was run for nine sessions on average. Since the procedure did not influence learning—when presented with the two targets, the fish did not show any preference in advance—we reduced this procedure to two sessions for Fish 3 and afterwards eliminated it completely. Note that for Fish 3 in the second, control experiment, we also conducted the pre-experiment learning phase in order to demonstrate that it is actually unnecessary. There was no difference between this additional replication with Fish 3 in the control experiment (Experiment 2) and the other fish in the control experiment with no learning phase.

In both phases of the experiment, spitting at one color resulted in one food pellet given only to the acting fish, while spitting at the other color resulted in one food pellet given to the acting fish and another food pellet given to its passive tank-mate. Hence, regardless of which target was hit, the outcome for the acting fish was the same, while the tank-mate only received a food pellet if a particular target was hit. In a case where both fish received food, the acting fish received its food pellet first.

Each fish in the experiment performed 15 sessions in the first phase. In the second phase, a switching point was defined when the fish spat at the new equal (social) color for more than 51% of the trials for three sessions in a row. The experiment then continued for an additional 25 sessions, including those three sessions. Fish 3 was required to stop after 19 sessions because of technical problems.

#### Experiment 2 - Control experiment

In the control experiment, only one fish was in the fish tank. Spitting at one color resulted in one food pellet being given to the fish, while spitting at the other color resulted in two food pellets being given, one to the acting fish and another one dropped into the empty side of the partition. To prevent food accumulation on the empty side of the tank, we used a small net to collect food from the empty side after each trial, even when no food pellet was given. The net was used after every spit (even if no food was delivered to the empty side of the partition) in order to prevent a differential effect on the behavior of the fish in the two conditions. As in the first experiment, in the control experiment the acting fish received the food first (see Supplementary Movie 2).

In the control experiment, two fish performed 15 sessions in the first phase. To ensure that the results, showing no difference in fish preference, were not due to the small number of sessions, an additional fish performed 25 sessions in the first phase (this number was determined by the number of sessions collected after the switch in the social experiment). For all fish, the second phase entailed 30 sessions to ensure there was no preference.

#### Experiment 3

In the unequal reward distribution experiment, two fish were in the dual tank. Spitting on one target resulted in one food pellet given to the acting fish only (1/0, self-advantageous), while spitting on the other color target resulted in one food pellet given to the acting fish and two food pellets given to its tank-mate (1/2, self-disadvantageous). As in the first experiment, the outcome for the acting fish was kept constant, regardless of which target was hit. Note that for two fish from the first experiment (Fish 3 and Fish 4), this procedure came after the first experiment, such that the outcome for the equal distribution (prosocial) target changed to the passive fish’s advantage. One fish was run especially for this experiment and performed only the first social phase, without the reversal phase.

#### Statistics and reproducibility

For the statistical analysis, in each phase we calculated the proportion of each target color choice in each session and then used one-sample *t*-test to compare between the choices made by the fish to chance. Each session contained 40 trials. The fish did not necessarily spit in all 40 trials; hence the proportion was calculated only for valid trials (i.e., from the sum of both colors). If a fish did not spit in more than half the trials within a session, we omitted that session. See Supplementary Note 7. We also calculated Bayes factors (BF) using the free software JASP (https://jasp-stats.org). See also Supplementary Note 11 for Wilcoxon signed rank analyses and Supplementary Note 9. Sample size. We adopted a sample size that has been frequently utilized in previous archerfish studies (see refs. ^37–39,41,48,56^). A total of six fish participated in the first experiment. A total of four fish participated in the second experiment; however, one did not meet the criterion for accurate hits and therefore was not included in the main analysis, see Supplementary Note 2 for its results that resemble the other fish. A total of three fish participated in the third experiment. See Supplementary Table 1 in the supplemental material for the role of each fish in the experiment, see also^79^.

### Reporting summary

Further information on research design is available in the Nature Portfolio Reporting Summary linked to this article.

### Supplementary information

Supplementary Information

Description of Additional Supplementary Files

Supplementary Movie 1

Supplementary Movie 2

Reporting Summary

## Data Availability

The data that support the findings of this study are available at the following link: 10.17605/OSF.IO/26YFJ.
