# Supplementary material for: Prosocial behavior in competitive fish: the case of the archerfish
Source: Commun Biol. 2023 Aug 8;6:822. doi: 10.1038/s42003-023-05195-1 (PMC10409803; doi:10.1038/s42003-023-05195-1)
Supplement: Supplementary file 1 — Supplementary Information [file 42003_2023_5195_MOESM1_ESM.pdf]

Supplementary Materials

Prosocial Behavior in Competitive Fish: The Case of the Archerfish

Orit Nafcha*, Dana Vilker, Simone Shamay-Tsoory, Shai Gabay

	**Pro-social (1/0 – 1/1) n=4**	**Control (1/0 – 1/1) n =3**	**Unequal (1/0 – 1/2) n =3**
**Fish 1**	With an additional passive fish	Presented in the control experiment as fish a	-
**Fish 2**	With fish 3 as a receiver	-	-
**Fish 3**	With fish 2 as a receiver	Presented in the control experiment as fish b	Presented in experiment 3 as fish a. with fish 2 as a receiver
**Fish 4**	With an additional passive fish	Presented in the control experiment as fish c	Presented in experiment 3 as fish b. with an additional passive fish
**Fish 5**	-	-	Presented in experiment 3 as fish c. with an additional passive fish

Supplementary Table 1: The role of each fish in the experiment

Supplementary Note 1: Descriptive statistics

	Phase 1 -*M(SD)*		Phase 2-*M(SD)*	
Fish number	P (equal, pro- social)	P (non-prosocial)	P (equal, pro- social)	P (non-prosocial)
Fish 1 (Fig. 2a):	0.69(0.12)	0.31(0.12)	0.63(0.1)	0.37(0.1)
Fish 2 (Fig. 2b):	0.66 (0.16)	0.34 (0.16)	0.6(0.06)	0.4(0.06)
Fish 3 (Fig. 2c)	0.85 (0.13)	0.15 (0.13)	0.74(0.11)	0.26(0.11)
Fish 4 (Fig. 2d)	0.67 (0.09)	0.33 (0.09)	0.61 (0.08)	0.39 (0.08)

Supplementary Table 2: Descriptive statistics - Experiment 1 - prosocial experiment.

Means and standard deviations for each fish, phase in the first (pro-social) experiment.

The following is a summary of the descriptive statistic for the all fish together for each target type selection in each phase. Phase 1: Prosocial (*M*=0.71, *SD*=0.08), Non-prosocial (*M*=0.28, *SD*=0.08); Phase 2: Prosocial (*M*=0.64, *SD*=0.06), Non-prosocial (*M*=0.35, *SD*=0.06).

	Phase 1 - *M(SD)*		Phase 2 - *M(SD)*	
Fish number	p(dual events)	p(single event)	p(dual events)	p(single event)
Fish 1 (Fig. 3a):	0.51(0.08)	0.48(0.08)	0.46(0.04)	0.54(0.04)
Fish 2 (Fig. 3b):	0.47 (0.08)	0.53 (0.08)	0.49(0.07)	0.51(0.07)
Fish 3 (Fig. 3c)	0.5 (0.08)	0.5(0.08)	0.47 (0.09)	0.53(0.09)

Supplementary Table 3: **Descriptive statistics- Experiment 2- control.**

Means and standard deviations for each fish, phase in the second (control) experiment.

The following is a summary across all fish for each target type selection in each phase: Phase 1 – dual events (*M* =0.5, *SD* =0.023), single event (*M* =0.5, *SD* =0.023); Phase 2 – dual events (*M*=0.473, *SD*=0.04), single event (*M* =0.527, *SD* =0.04).

	Ten last trials of the social exp.		unequal exp.			
	M(SD)		M(SD)			
			Analysis of all sessions		Analysis from switch	
Fish number	P (equal, pro-social)	P(non-prosocial)	P (1/2 Social unequal)	P (1/0, non-prosocial)	P (1/2 Social unequal)	P (1/0, non-prosocial)
Fish 1 (Fig. 4a):	0.75(0.14)	0.25(0.14)	0.47(0.15)	0.53(0.15)	0.44(0.13)	0.56(0.13)
Fish 2 (Fig. 4b):	0.67 (0.08)	0.33(0.08)	0.48(0.1)	0.52(0.1)	0.47(0.01)	0.53(0.01)
Fish 3 (Fig. 4c)	0.6 (0.08)	0.4 (0.08)	0.4(0.08)	0.6(0.08)	0.39(0.08)	0.61(0.08))

Supplementary Table 4: Descriptive statistics - Experiment 3: the unequal reward distribution experiment. Means and standard deviations for each fish, phase in the third experiment.

The following is a summary of descriptive statistics across all fish for each target type selection for:

The ten last trials from the social (1/1) phase: social targets (1/1) (*M*=0.67, *SD*=0.069); non-prosocial targets (1/0) (*M*=0.33, *SD*=0.069).

All sessions after changing the mapping for the social target so 1/2 – self-disadvantage/other advantage targets (1/2) (*M*=0.45, *SD*=0.045); self-advantage targets (1/0) (*M*=0.55, *SD*=0.045).

The sessions from the switch: self-disadvantage targets (1/2) (*M*=0.43, *SD*=0.03), self-advantage targets (1/0) (*M*=0.57, *SD*=0.03).

Supplementary Note 2: Another replication of the control experiment.

A fourth fish participated in the control experiment but did not meet the criterion for accurate hits. When considering a wider radius, we found a pattern of results similar to that of the other control fish. That is, the fish showed no preference for any of the color targets: Phase 1- $t_{(19)}$=1.34 , *p* = 0.195, *d*=0.3, *BF10*=0.507, [Average p(dual events)= 0.48(0.05), Average p(single event)=0.51(0.05)]; Phase 2- $t_{(29)}$=.18 , *p*= 0.85, *d*= 0.03, *BF10*=0.19, [Average p(dual events)= 0.49(0.08), Average p(single event)=0.5(0.08)].

Supplementary Note 3: Comparing the social to the control experiment (phase 1).

Analyses between the first phase of the first experiment and the second experiment. Since in the second phase there was also a reversal learning period, we compare only the first phase between the experiments. Fish 1,3, and 4 from the first social experiment also conducted the control experiment (see Table 1 above). There was a significant effect between those phases for each fish: fish 1 social and in the control: *t*(14)=4.7, *p*<0.001, *d*=1.233, *BF10*=111.06 (1 way 222.05); fish 3 social and b in the control- *t(*14)=8.24, *p*<0.001, *d*= 2.12, *BF10*=17959.9; fish 4(social)and c(control)- *t*(14)=6.03, *p*<0.001, *d*= 1.55, *BF10*=808.6.

Over all descriptive mean and SD for the control vs. social comparison is *M*= 0.737 (*SD*=0.09) for the choice in the social target *M*=0.497 (*SD*=0.02) in the control phase for choosing the 2 food pellets with no fish on the other side.

Supplementary Note 4.

Analyses of all the data including the trials before the pre-defined reversal point for all fish-phase 2: *t* (144) =3.73*, p*<0.001, *d*= 0.3 *BF10*=62.63

Supplementary Note 5: Color preference

Stimuli were presented using E-prime 2 software. Each trial began with the flickering of two black location markers presented randomly in one of four possible locations (front left and right and back left and right). The markers appeared for 200ms at a time, with a 600ms interval between appearances. After the location markers disappeared, two color targets appeared for 5500ms or until the fish responded. The targets appeared at the same location as the black location markers. After the targets disappeared, a blank interval screen was presented between trials for 5500ms. During this time, the fish received a food pellet regardless of which target it chose. The position of each target's color within the pair was counterbalanced.

Allocation of colors in each phase for each fish:

Note that for all fish in experiments one and two, the colors were switched in the second phase.

Experiment 1: The prosocial experiment.				
	Phase 1		Phase 2	
Fish number	Equal, pro-social	Non-prosocial	Equal, pro-social	Non-prosocial
Fish 1	Black	Red	Red	Black
Fish 2	Black	Green	Green	Black
Fish 3	Black	Red	Red	Black
Fish 4	Green	Black	Black	Green

Supplementary Table 5. Color assignment in the first prosocial experiment.

Experiment 2: Control				
	Phase 1		Phase 2	
Fish number	Single event	Dual event	Single event	Dual event
Fish a	Green	Blue	Blue	Green
Fish b	Green	Blue	Blue	Green
Fish c	Black	Green	Green	Black

Supplementary Table 6. Color assignment in the second, control, experiment.

In order to rule out that the fish cannot distinguish between the color green and blue please see note 6 and also please note that the colors used for Fish c were the same as it was for it in the social experiment (fish 4) in order to test whether the results of the other fish were dependent on the specific color properties employed.

Experiment 3: The unequal reward distribution experiment.				
	Pre-experiment		The experiment	
Fish number	Equal, pro-social	Non-prosocial	Social unequal (1/2)	Non-prosocial
Fish a	Red	Black	Red	Black
Fish b	Black	Green	Black	Green
Fish c	Green	Black	Green	Black

Supplementary Table 7. Color assignment in the third, unequal reward distribution experiment.

Supplementary Note 6: Distinguishing between green and blue.

Despite evidence indicating that fish have color vision similar to that of humans [1, 2], we conducted a control experiment in order to rule out the possibility that the fish cannot distinguish between blue and green—the colors allocated to the control experiment.

In this experiment, one fish in a single tank was required to choose between a blue target, which resulted in receiving one food pellet, and a green target, which resulted in no food reward. We found a significant bias toward the blue target: $t_{(9)} = 4.09$, p=0.003, d= 1.29, *BF10*= 17.87, Average p(blue)= 0.61(0.09), Average p(green)=0.38 (0.09).

Supplementary Note 7: Filtering rule.

Sessions in which the fish did not spit during more than 20 trials (half of the trials) were removed from the analysis. In total, in the first experiment, two sessions were removed for Fish 1 in the second phase and 11 sessions for Fish 4 in the first phase. In the second, control, experiment, one session was removed for Fish 2 in the first phase. In the third experiment, one session was removed for Fish 2. Six sessions were removed for Fish 3 in the first phase (the first-pro-social phase) and four sessions in the third experiment.

Supplementary Note 8: The pattern of results for each fish in the first session of the prosocial experiment.

In order to present the pattern of results in the first session, we calculated a moving average (with a window of five trials) for choosing the non-prosocial target. This analysis indicates two points: a) a more detailed pattern of preferences in the first introduction to the task, showing that there was exploration at the beginning with sampling of the non-social target that diminished as the session continued; b) an overall bias toward the prosocial target, mostly

manifesting in the second half of the first session, indicating that it was not hard for the fish to learn the difference between the target outcomes.

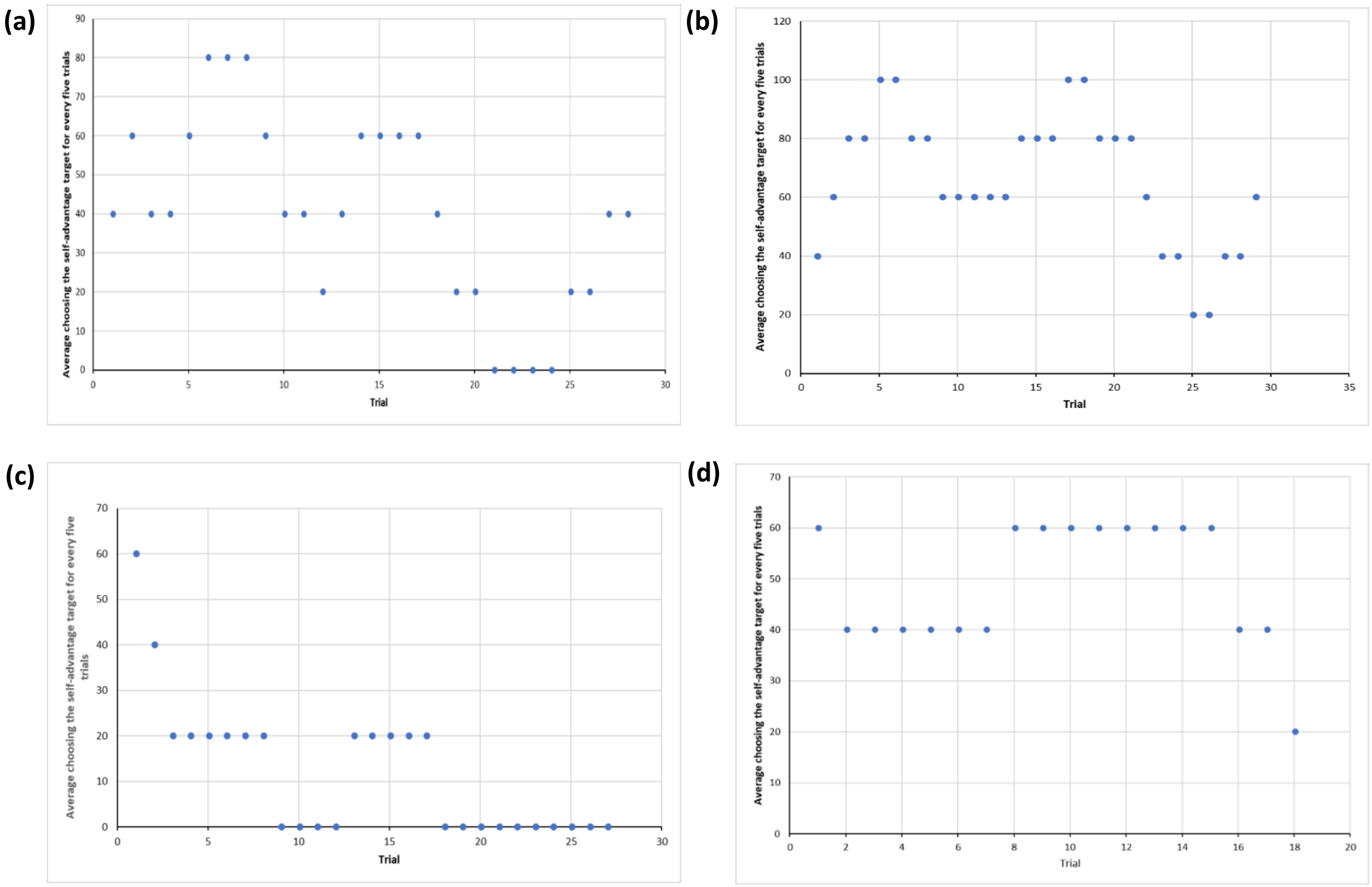

Supplementary Figure 1. A moving average of the fish choices in the non-prosocial target at the first session. We calculated a moving average in the first session for each fish (i.e., calculated the percentages for each five choices throughout the first session). The order of the figures is similar to Figure 2 in the article.

Supplementary Note 9: Experimenter bias limitation/concerns. The experimenter could not be blind to the different mapping rewards since she was the one delivering the food. However, the results of Fish 4 in the first, prosocial experiment, the results of the third fish in the control experiment, and the results of two fish in the third experiment were coded by a naive research assistant who was blind to the conditions (she was instructed to observe only

which color the fish spat at and not to look at the subsequent outcome). In addition, the same research assistant coded 30% of all the remaining videos. A comparison of her analysis to those of the original coder (the experimenter) yielded identical results, demonstrating perfect inter-judge reliability.

Supplementary Note 10: Group analyses.

(One sample t-test, two way, test value=0.5)

Experiment 1 (prosocial): Phase 1: $t(3) = 5.027$, $p = 0.015$, $d = 2.513$, (prosocial>non-prosocial); Phase 2: $t(3) = 4.203$, $p = 0.025$, $d = 2.102$;(prosocial>non-prosocial).

Experiment 2 (control): Phase 1:$t(2) = -0.32$, *n.s.*, $d = -0.18$; Phase 2: $t(2) = -3.2$, *n.s.*, $d = -1.85$,

Experiment 3 (unequal): Ten last trials (prosocial is 1/1): $t(2) = 4.38$ $p < 0.05$, $d = 2.53$ (prosocial>non-prosocial); all data : $t(2) = -1.8$, *n.s.*, $d = 1.06$; After the switch:): $t(2) = -3$, $p = 0.09$ $d = 1.76$ (non-prosocial>prosocial).

Supplementary Note 11: Wilcoxon signen rank analyses for the prosocial target (one sample, 0.5)

Experiment 1 (prosociality):

Fish 1: phase 1 ($V = 105$, $p = 0.001$); phase 2 ($V = 325$, $p < 0.001$)

Fish 2: phase 1 ($V = 120$, $p < 0.001$); phase 2 ($V = 171$, $p < 0.001$)

Fish 3: phase 1 ($V = 120$, $p < 0.001$); phase 2 ($V = 300$, $p < 0.001$)

Fish 4: phase 1 ($V = 119$, $p < 0.001$); phase 2 ($V = 316$, $p < 0.001$)

Experiment 2 (control):

Fish a: phase 1 ($V = 170$, $p = 0.33$); phase 2 ($V = 43$, $p < 0.001$ (1 food>2food))

Fish b: phase 1 ($V=38$, $p=0.22$); phase 2 ($V=161$, $p=0.35$)

Fish c: phase 1 ($V=52$, $p=1$); phase 2 ($V=137$, $p=0.08$ (1 food>2food))

Experiment 3 (unequal):

Fish a: 10 last (social is 1/1) ($V=45$, $p=0.009$); all data ($V=295$, $p=0.19$); after the switch- ($V=135$, $p=0.017$)

Fish b: 10 last (social is 1/1) ($V=55$, $p=0.002$); all data ($V=402$, $p=0.28$); after the switch ($V=264$, $p=0.08$ (non-prosocial>social))

Fish c: 10 last (social is 1/1) ($V=54$, $p=0.004$); all data ($V=16$, $p<0.001$); after the switch ($V=520$, $p=0.001$ (non-prosocial>social))

Supplementary Movie 1. A video presentation demonstrating the prosocial experiment set-up, the two fish in the dual tank, and the performance of the acting fish on two pro-social trials and two non-prosocial trials.

Supplementary Movie 2. A video presentation demonstrating the control experimental set-up, the single fish in the dual tank, and the performance of the fish in a dual-event trial and in a single-event trial.

Supplementary References

1. Newport, C. & Schuster, S. Archerfish vision: Visual challenges faced by a predator with a unique hunting technique. *Seminars in Cell and Developmental Biology* (2020) doi:10.1016/j.semcdb.2020.05.017.

2. Temple, S., Hart, N. S., Marshall, N. J. & Collin, S. P. A spitting image: specializations in archerfish eyes for vision at the interface between air and water. *Proc. R. Soc. B Biol. Sci.* **277**, 2607–2615 (2010).
