# Supplementary material for: Prosocial behavior in competitive fish: the case of the archerfish
Source: Commun Biol. 2023 Aug 8;6:822. doi: 10.1038/s42003-023-05195-1 (PMC10409803; doi:10.1038/s42003-023-05195-1)
Supplement: Supplementary file 2 — Description of Additional Supplementary Files [file 42003_2023_5195_MOESM2_ESM.pdf]

Description of Additional Supplementary Files

File name: Supplementary Movie 1
Description: The prosocial task.
A video presentation demonstrating the prosocial experiment set-up, the two fish in the dual tank, and the performance of the acting fish on two pro-social trials and two non-prosocial trials.

File name: Supplementary Movie 2
Description: The control task.
A video presentation demonstrating the control experimental set-up, the single fish in the dual tank, and the performance of the fish in a dual-event trial and in a single-event trial.
