# Supplementary material for: Prosocial behavior in competitive fish: the case of the archerfish
Source: Commun Biol. 2023 Aug 8;6:822. doi: 10.1038/s42003-023-05195-1 (PMC10409803; doi:10.1038/s42003-023-05195-1)
Supplement: Supplementary file 5 — Reporting Summary [file 42003_2023_5195_MOESM5_ESM.pdf]

nature portfolio

Corresponding author(s): Orit Nafcha

Last updated by author(s): 19/07/2023

Reporting Summary

Nature Portfolio wishes to improve the reproducibility of the work that we publish. This form provides structure for consistency and transparency in reporting. For further information on Nature Portfolio policies, see our Editorial Policies and the Editorial Policy Checklist.

Statistics

For all statistical analyses, confirm that the following items are present in the figure legend, table legend, main text, or Methods section.

n/a	Confirmed	
☐	☒	The exact sample size (*n*) for each experimental group/condition, given as a discrete number and unit of measurement
☐	☒	A statement on whether measurements were taken from distinct samples or whether the same sample was measured repeatedly
☐	☒	The statistical test(s) used AND whether they are one- or two-sided<br>*Only common tests should be described solely by name; describe more complex techniques in the Methods section.*
☒	☐	A description of all covariates tested
☒	☐	A description of any assumptions or corrections, such as tests of normality and adjustment for multiple comparisons
☐	☒	A full description of the statistical parameters including central tendency (e.g. means) or other basic estimates (e.g. regression coefficient) AND variation (e.g. standard deviation) or associated estimates of uncertainty (e.g. confidence intervals)
☐	☒	For null hypothesis testing, the test statistic (e.g. *F*, *t*, *r*) with confidence intervals, effect sizes, degrees of freedom and *P* value noted<br>*Give P values as exact values whenever suitable.*
☒	☐	For Bayesian analysis, information on the choice of priors and Markov chain Monte Carlo settings
☒	☐	For hierarchical and complex designs, identification of the appropriate level for tests and full reporting of outcomes
☐	☒	Estimates of effect sizes (e.g. Cohen's *d*, Pearson's *r*), indicating how they were calculated

Our web collection on statistics for biologists contains articles on many of the points above.

Software and code

Policy information about availability of computer code

Data collection	Stimuli were presented on a computer screen (21-inch Samsung LCD monitor (model S24C650PL)) using E-prime 2 software. The data were recorded using a GigE Camera color (120 fps 640 × 480 1/4) and a GoPro HERO7.
Data analysis	The data were analyzed using the software Statistica (StatSoft) and the free software JASP (https://jasp-stats.org).

For manuscripts utilizing custom algorithms or software that are central to the research but not yet described in published literature, software must be made available to editors and reviewers. We strongly encourage code deposition in a community repository (e.g. GitHub). See the Nature Portfolio guidelines for submitting code & software for further information.

Data

Policy information about availability of data

All manuscripts must include a data availability statement. This statement should provide the following information, where applicable:

- Accession codes, unique identifiers, or web links for publicly available datasets
- A description of any restrictions on data availability
- For clinical datasets or third party data, please ensure that the statement adheres to our policy

Data and materials availability: The data that support the findings of this study are available at the following link: https://doi.org/10.17605/OSF.IO/26YFJ

Human research participants

Policy information about studies involving human research participants and Sex and Gender in Research.

Reporting on sex and gender	*Use the terms sex (biological attribute) and gender (shaped by social and cultural circumstances) carefully in order to avoid confusing both terms. Indicate if findings apply to only one sex or gender; describe whether sex and gender were considered in study design whether sex and/or gender was determined based on self-reporting or assigned and methods used. Provide in the source data disaggregated sex and gender data where this information has been collected, and consent has been obtained for sharing of individual-level data; provide overall numbers in this Reporting Summary. Please state if this information has not been collected. Report sex- and gender-based analyses where performed, justify reasons for lack of sex- and gender-based analysis.*
Population characteristics	*Describe the covariate-relevant population characteristics of the human research participants (e.g. age, genotypic information, past and current diagnosis and treatment categories). If you filled out the behavioural & social sciences study design questions and have nothing to add here, write "See above."*
Recruitment	*Describe how participants were recruited. Outline any potential self-selection bias or other biases that may be present and how these are likely to impact results.*
Ethics oversight	*Identify the organization(s) that approved the study protocol.*

Note that full information on the approval of the study protocol must also be provided in the manuscript.

Field-specific reporting

Please select the one below that is the best fit for your research. If you are not sure, read the appropriate sections before making your selection.

☐ Life sciences ☒ Behavioural & social sciences ☐ Ecological, evolutionary & environmental sciences

For a reference copy of the document with all sections, see nature.com/documents/nr-reporting-summary-flat.pdf

Behavioural & social sciences study design

All studies must disclose on these points even when the disclosure is negative.

Study description	Quantitative
Research sample	Overall six archerfish (Toxotes chatareus) participated in the experiment. Note that the fish in the first and the third studies shared the dual tank with an additional passive fish, with a partition between them.
Sampling strategy	Fish were randomly chosen from a pull of fish available for experimentation in the lab. The sample size used in this study is acceptable in the field (see, e.g., Newport et al., 2016; Leadner et al., 2021; Saban et al., 2017; Schlegel & Schuster, 2008). It should also be noted that each fish completed thousands of trials in a period of 8-9 months.
Data collection	Stimuli were presented on a computer screen (21-inch Samsung LCD monitor (model S24C650PL)) using E-prime 2 software. The data were recorded using a GigE Camera color (120 fps 640 × 480 1/4) and a GoPro HERO7. The experimenter could not be blind to the different mapping rewards since she was the one delivering the food. However, the results of Fish 4 in the first experiment, the results of the third fish in the control experiment, and the results of two fish in the third experiment were coded by a naive research assistant who was blind to the conditions (she was instructed to observe only which color the fish spat at and not to look at the subsequent outcome). In addition, the same research assistant coded 30% of all the remaining videos. A comparison of her analysis to those of the original coder (the experimenter) yielded identical results, demonstrating perfect inter-judge reliability.
Timing	The overall data collection started in 2017 and ended in 2020. Note that there were no gaps during this period.
Data exclusions	Sessions in which the fish did not spit during more than 20 trials (half of the trials) were removed from the analysis. In total, in the first experiment, two sessions were removed for Fish 1 in the second phase and 11 sessions for Fish 4 in the first phase. In the second, control, experiment, one session was removed for Fish 2 in the first phase. In the third experiment, one session was removed for Fish 2. Six sessions were removed for Fish 3 in the first phase (the pro-social study) and four sessions in the third experiment.<br>In addition, in the control experiment one fish did not meet the criterion for accurate hits and therefore was not included in the main analysis. Yet note that the results for this fish, which are presented in the supplemental material, resembled those of the other control fish.
Non-participation	N/A
Randomization	The fish were not allocated to separate groups.

Reporting for specific materials, systems and methods

We require information from authors about some types of materials, experimental systems and methods used in many studies. Here, indicate whether each material, system or method listed is relevant to your study. If you are not sure if a list item applies to your research, read the appropriate section before selecting a response.

Materials & experimental systems

n/a	Involved in the study
☒	☐ Antibodies
☒	☐ Eukaryotic cell lines
☒	☐ Palaeontology and archaeology
☐	☒ Animals and other organisms
☒	☐ Clinical data
☒	☐ Dual use research of concern

Methods

n/a	Involved in the study
☒	☐ ChIP-seq
☒	☐ Flow cytometry
☒	☐ MRI-based neuroimaging

Animals and other research organisms

Policy information about studies involving animals; ARRIVE guidelines recommended for reporting animal research, and Sex and Gender in Research

Laboratory animals	The study did not involve laboratory animals.
Wild animals	Species: Spotted Archerfish (Toxotes chatareus)<br>Age: Unknown. All individuals have been caught in the wild and acquired through pet trade. Ages likely range between one to eight years.<br>The fish were purchased from local suppliers (e.g., Aquazone LTD).
Reporting on sex	Sex: Unknown. The sex of individual T. chatareus cannot be externally identified.
Field-collected samples	Housing: Fish were housed in a dual tank that was separated by a transparent partition. Each tank was 55 L aquarium partially filled (water volume approximately 35 L) with brackish water (salinity 10 ppt), and kept at water temperature of approximately 28 degrees Celsius and room temperature of 24 degrees Celsius. All aquariums contain gravel aquarium substrate, a water heater, and a water filter.
Ethics oversight	All our data were collected and approved by the University of Haifa's animal ethical committee and the State of Israel's laws on animal care and experimentation.

Note that full information on the approval of the study protocol must also be provided in the manuscript.
